# Psychometric properties of the Postgraduate Hospital Educational Environment Measure in an Iranian hospital setting

**DOI:** 10.3402/meo.v19.24546

**Published:** 2014-08-08

**Authors:** Shahrzad Shokoohi, Amir Hossein Emami, Aeen Mohammadi, Soleiman Ahmadi, Rita Mojtahedzadeh

**Affiliations:** 1 Department of Medical Education, Virtual School and Medical School, Tehran University of Medical Sciences, Tehran, Iran; 2 Department of E-learning in Medical Education, Virtual School, Tehran University of Medical Sciences, Tehran, Iran; 3 Department of Medical Education, Shaheed Beheshti University of Medical Sciences, Tehran, Iran

**Keywords:** education environment, postgraduate, evaluation, psychometrics

## Abstract

**Background:**

Students’ perceptions of the educational environment are an important construct in assessing and enhancing the quality of medical training programs. Reliable and valid measurement, however, can be problematic – especially as instruments developed and tested in one culture are translated for use in another.

**Materials and method:**

This study sought to explore the psychometric properties of the Postgraduate Hospital Educational Environment Measure (PHEEM) for use in an Iranian hospital training setting. We translated the instrument into Persian and ensured its content validity by back translation and expert review prior to administering it to 127 residents of Urmia University of Medical Science.

**Results:**

Overall internal consistency of the translated measure was good (*a*=0.94). Principal components analysis revealed five factors accounting for 52.8% of the variance.

**Conclusion:**

The Persian version of the PHEEM appears to be a reliable and potentially valid instrument for use in Iranian medical schools and may find favor in evaluating the educational environments of residency programs nationwide.

Since knowledge, thinking, and learning are context dependent (1), it is important to acknowledge the relationship between students’ educational environment and their academic achievement and satisfaction (2). Clinical education, in particular, is an environment with unique challenges and an effective clinical teaching environment balances and integrates the relevancy of professional education to patients as well as students’ active participation, professional thinking, and behaviors (3). Assessing the quality of the clinical learning environment, then, should be done periodically. However, the reliability and validity of the resulting scores should first be established in the setting the instrument is to be used.

The Postgraduate Hospital Educational Environment Measure (PHEEM) was developed and validated in Scotland and the West Midlands using grounded theory and Delphi methods (4).

PHEEM is a 40-item questionnaire with three subscales tapping respondents’ perceptions of: 1) role autonomy; 2) teaching; and 3) social support. Each item is measured on a five-point Likert-type scale, and is scored: 4 for ‘Strongly Agree’, 3 for ‘Agree’, 2 for ‘Uncertain’, 1 for ‘Disagree’, and 0 for ‘Strongly Disagree’ (four items are reverse-coded prior to scoring). Total scores range from 0 to 160, with higher scores indicating a higher quality educational climate (4).

While researchers in different countries have successfully translated and analyzed the psychometric features of the PHEEM (3, 5–10), its validity in an Iranian training context is unknown. Thus, this study assesses the psychometric properties of Persian (Farsi) version of the PHEEM in an Iranian residency program setting.

## Methods

After garnering the author's permission to adapt the PHEEM, an Iranian physician proficient in English translated the instrument into Persian. We then sent the translated version to six reviewers (three clinical teachers and three medical education experts) who were asked to assess item relevance for Iranian residency programs. Based on their comments, we made minor modifications to several items. To make the wording more appropriate in an Iranian context, item #7 was changed from *There is racism in this post* to *Discrimination exists here* and item #17 was changed from *My hours conform to the New Deal* to *My working hours are in line with official rules of my country*. The edited questionnaire was then back-translated to English by a professional translator, who was blinded to the original version. Another translator subsequently reviewed the back-translated version alongside the original questionnaire and found no conceptual differences. Finally, 35 randomly selected residents in Urmia University of Medical Sciences completed the Persian version to assess its face validity and reliability. Spaces for written suggestions were provided after each item; however, no comments were made regarding item exclusion or obscurity.

The pre-tested questionnaire was then administered to 170 residents of the university. Construct validity of the scores was assessed using a principal components analysis (PCA) and a Varimax rotation. We considered eigenvalues ≥1.6 and factor loadings ≥0.3; a visual inspection of the inflection point in the scree plot dictated the factor extraction criteria. All analyses were conducted using SPSS Version 16.0 (11), and a critical p-value of ≤0.05 was set for all inferential analyses.

## Results

One hundred and twenty-eight (128) residents from seven clinical departments (anesthesiology, cardiology, internal medicine, surgery, obstetrics and gynecology, pathology, and pediatrics) returned completed questionnaires. The corrected response rate was 74.7% (one returned instrument with < 50% of items completed was excluded from the study). Participants consisted of 52 women and 75 men, with a mean age of 33 years. Per the original subscales, the internal consistency (Cronbach's α) was 0.94 for 40 items and 0.84, 0.90, and 0.80 for the role autonomy, teaching, and social support subscales, respectively.

A PCA of residents’ responses revealed five factors with eigenvalues ≥1.6, accounting for 52.8% of total variance. Items loading on multiple factors were assigned based on the largest coefficient. Extracted factors were identified by two researchers (Table 1).

**Table 1:** Comparison of factors extracted in the study and the original questionnaire

	Factor	Name	Items	Cronbach's α
Study subscales	1	Teaching	*10, 15, 22, 28*, 30, *31, 33*, 35, *37*, *39*, 40	0.88
	2	Education system	1, 2, 3, 4, 6, 9, 14, 21, 23	0.84
	3	Training facilities	17, 20, 26, 27, 32, 38	0.78
	4	Job satisfaction	12, 16, 18, 19, 29, 34, 36	0.79
	5	Social support	5, 7*, 8*, 11*, 13*, *24*, *25*	0.74
Original subscales	1	Perception of role autonomy	1, 4, 5, 8*, 9, 11*, 14, 17, 18, 29, 30, 32, 34, 40	0.84
	2	Perception of teaching	2, 3, 6, *10*, 12, *15*, 21, *22*, 23, 27, *28*, *31*, *33*, *37*, *39*	0.90
	3	Perception of social support	7*, 13*, 16, 19, 20, *24*, *25*, 26, 35, 36, 38	0.80

* Items reverse-coded prior to scoring.

Note: Persian version of the questionnaire can be accessed at: http://etums.tums.ac.ir/Default.aspx?PageID=166.

As shown in Table 1, 8 of 11 items (in italics) loading on Factor 1, ‘Teaching’, were part of the ‘Perception of Teaching’ subscale of the original questionnaire. Four of seven items loaded similarly on ‘Social Support’. A summary of factor loadings ≥ 0.30 is presented in Table 1.

## Discussion

Our psychometric analysis of the Persian version of the PHEEM is encouraging. In the assessment by a group of experts (clinical teachers and medical educators), the instrument showed acceptable content validity, with only two items (items 7 and 17) requiring changes for use in an Iranian hospital setting. In pretesting with residents, the instrument also had suitable face validity. Regarding construct validity, the PCA revealed a multi-dimensional structure. The loading of items related to clinical teaching quality in the first factor reflects residents’ perceptions of the educational environment. Both the original and the revised instruments showed good internal consistency across the representative subscales. Numerous studies, in accordance with our own, have reported high internal consistency for the PHEEM (3, 5–10). Our PCA results are similar to a Chilean study (9), in which the PHEEM was also found to tap five unique factors. Conversely, our results are disparate with studies showing the PHEEM to have a unidimensional factor structure (5, 6, 8–10).

Comparing the results of this study with the original questionnaire shows that most of ‘perception of role autonomy’ items in the original work loaded on three factors of our study: ‘education system’, ‘training facility’ and ‘job satisfaction’. Conversely, only four items of the original instrument's social support subscale loaded on our social support factor, with the remaining seven distributed to other factors. As the nature of knowledge, thinking, and learning is context specific, the cultural and educational system differences may have caused ‘role autonomy’ to be perceived as three separate dimensions. These contextual considerations may also underlie observed differences in the social support factor.

## Conclusion

The Persian (Farsi) version of the PHEEM shows promise as a reliable and potentially valid instrument for assessing the clinical educational environment in this context. We recommend expanded study of this instrument perhaps in combination with qualitative approaches to identify factors affecting differences in group perceptions of the educational environment.
